# Sudden unexpected death in epilepsy disclosure causes anxiety in patients with epilepsy: a Chinese questionnaire survey

**DOI:** 10.3389/fneur.2023.1284050

**Published:** 2023-11-14

**Authors:** Yuanhang Pan, Gengyao Hu, Zezhi Wang, Na Yuan, Zihan Wei, Xia Li, Xiaohua Hou, Jian Wang, Xinbo Zhang, Ze Chen, Shuyi Qu, Junxiang Bao, Yonghong Liu

**Affiliations:** ^1^Department of Neurology, Xijing Hospital, Fourth Military Medical University (Air Force Medical University), Xi’an, China; ^2^Department of Neurology, Xian Children’s Hospital, Xi’an, China; ^3^Department of Neurology, First Affiliated Hospital of Harbin Medical University, Harbin, China; ^4^Department of Neurosurgery, Tangdu Hospital, Fourth Military Medical University (Air Force Medical University), Xi’an, China; ^5^Department of Aerospace Hygiene, Fourth Military Medical University (Air Force Medical University), Xi’an, China

**Keywords:** sudden unexpected death in epilepsy, epilepsy, survey, anxiety, counsel

## Abstract

**Background and objective:**

Sudden unexpected death in epilepsy (SUDEP) has been regarded as a leading cause of premature death in patients with epilepsy (PWE). Although patients, relatives and caregivers have the right to be informed of SUDEP, neurologists prefer not to release the facts for fear of associated anxiety. In the study, a Chinese questionnaire survey was carried out to elucidate effect of SUDEP disclosure on anxiety in PWE and variables determining the anxiety of patients and provided suggestions for SUDEP disclosure.

**Methods:**

A survey study in China was conducted. We recruited 305 PWE from 3 tertiary epilepsy centers who attended outpatient clinic from December 2021 to February 2022. Two hundred and thirty-two PWE completed the screening evaluation, survey and Hamilton anxiety rating scale (HAMA) twice with 171 PWE completing third HAMA at follow-up. HAMA scores at baseline, T1, T2 were compared using analysis of variance and dependent samples *t*-test. The variables related to anxiety were screened out by univariate analysis and used for multivariate logistic regression.

**Result:**

We found 127 (54.7%) among the 232 participants experienced anxiety after SUDEP disclosure. HAMA scores at T1 were significantly higher than at baseline and T2, while there was no statistical difference between baseline and T2. Medical insurance, seizure severity, and whether the PWE supported SUDEP being disclosed to their relatives and caregivers only were associated with the occurrence of anxiety.

**Conclusion:**

SUDEP disclosures may cause short-term acute anxiety, but have no long-term effects in PWE. Acute anxiety caused by SUDEP disclosure may be more common in PWE with NCMI and severe seizures. Meanwhile, compared with indirect SUDEP disclosure to their relatives and caregivers, direct SUDEP disclosure to PWE reduces the risk of anxiety. Recommendations are provided to avoid anxiety caused by SUDEP disclosure.

## 1. Introduction

Epilepsy appears as a neurological disorder, characterized by recurrent and unprovoked seizures and by the neurobiologic, cognitive, psychological, and social consequences of this condition (1). Sudden unexpected death in epilepsy (SUDEP) has been reported and regarded as a leading cause of premature death in patients with epilepsy (PWE). Among all the neurologic diseases, SUDEP ranks secondly only to stroke in shaving the potential life expectancy (2). A Swedish national survey reported that the incidence rate of definite/probable SUDEP was 1.20/1000 patient-years (3). A few systematic reviews and meta-analyses have found that the pooled estimated incidence rate of SUDEP was 1.40/1000 patient-years. The incidence rate of SUDEP was 23 times higher than sudden death in the total population of the same age. Among 20–45 years-olds, the difference could be up to 27 times (4, 5). The health public burden of SUDEP has raised much attention of both neurologists and medical institutions.

The 2017 Practice Guide Summary published by the American Academy of Neurology and the American Epilepsy Society advocated neurologists notifying incidence, risk factors, and precautions of SUDEP to PWE (6). Nevertheless, the Practice Guide is not widely adopted by neurologists. Previous several investigations revealed 41.5% and 64% of neurologists seldom discussed SUDEP with PWE or caregivers (7–9) for fear of evoking anxiety (7, 8, 10). Anxiety is an important trigger for seizures in PWE. However, there are evidence suggesting the SUDEP disclosure not causing chronic anxiety but promoting medication compliance of epileptic patients (11, 12). Even more, PWE and caregivers have the right to be given the information (12, 13).

The present study aimed to evaluate the short- term and long-term effects of SUDEP disclosure on anxiety in PWE and identify determinants of anxiety after SUDEP disclosure. Based the result, a patient-centered communication strategy would be established which may amplify the proportion of SUDEP disclosure in PWE and their relatives.

## 2. Materials and methods

### 2.1. Participants

Totally, 305 PWE were recruited, who were followed up by Epilepsy Unit of Xijing hospital, Department of Neurology, First affiliated hospital of Harbin Medical University and Department of pediatric Neurology, Xi’an Children’s Hospital of P.R. China from December 2021 to February 2022, 232 PWE completed the screening evaluation and survey. The inclusion criterion was the established diagnosis of epilepsy. The exclusion criteria were as follow: prior SUDEP disclosure and awareness, cognitive dysfunction, diagnosed psychiatric comorbidities, subthreshold symptoms, meaning persons with higher scores in Hamilton anxiety rating scale (HAMA) that do not fulfill the criteria for the disorder, inability to complete survey.

**Figure 1:**
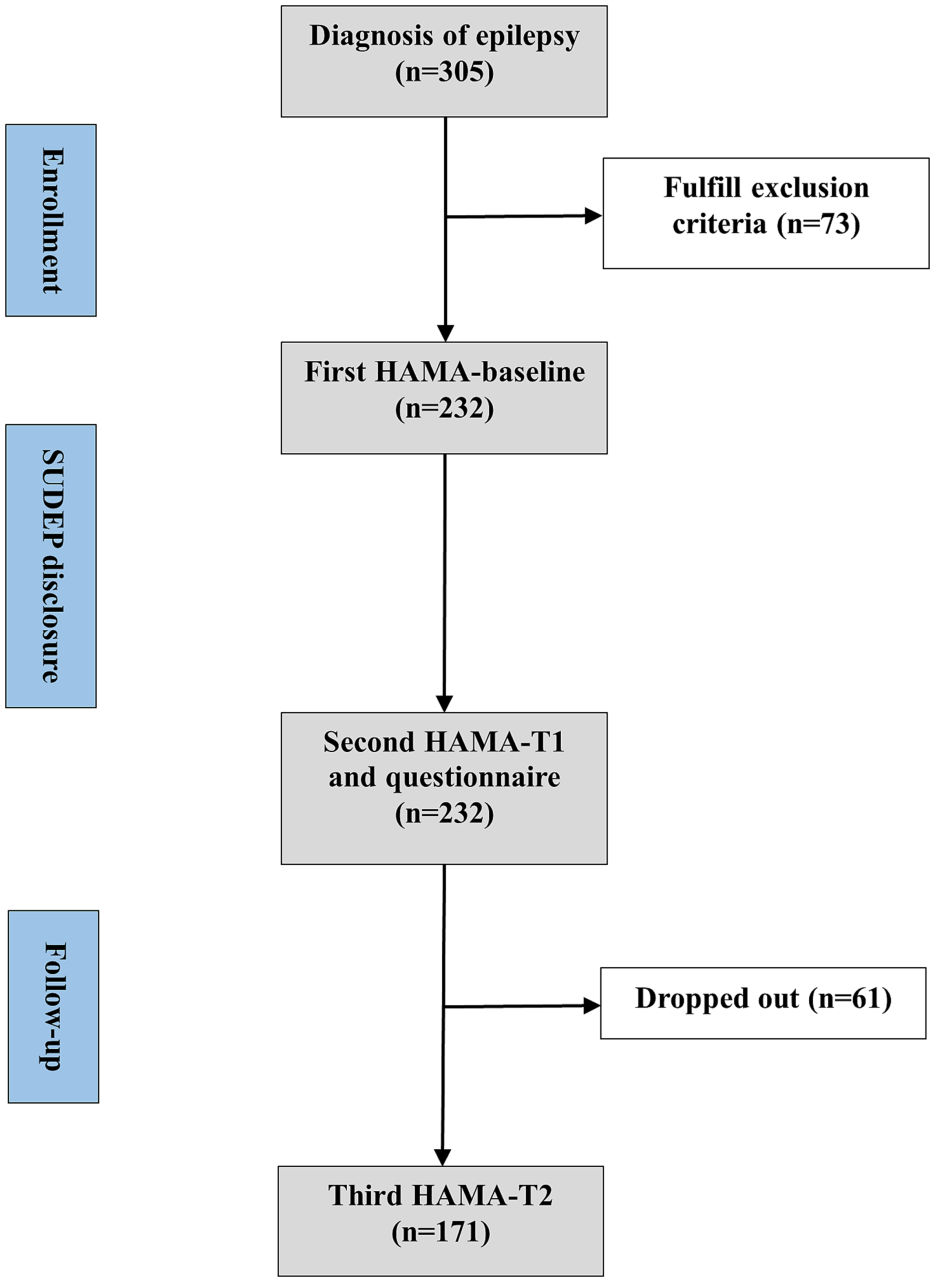
Experimental design.

**Figure 2:**
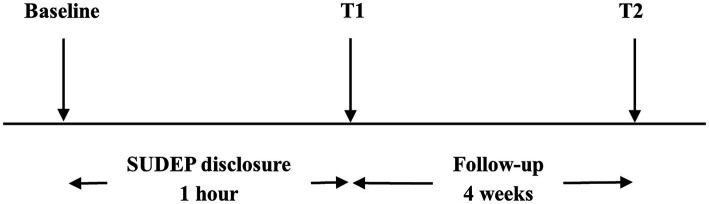
Flow chart.

Neurologists assessed the participants’ anxiety levels using the Hamilton anxiety rating scale (HAMA) before the SUDEP disclosure (baseline) and notified the participants of SUDEP-related information including individual risks and precautions. Within 1 h later, all the participants were invited to filled out the questionnaire and completed second HAMA accompanied by their caregivers under assistance of one neurologist during face-to-face interviews (T1). Once completing the questionnaire, participants and their caregivers could consult neurologist about the SUDEP further. They would also be presented with a handout on SUDEP after the interview. Four weeks later, the participants were invited to complete the third HAMA, either online or by telephone (T2). All the neurologists involved had been trained on skills of assisting participants to complete questionnaires. Experimental design is visualized in Figure 1. Flow chart is visualized in Figure 2.

The study was approved by the Medical Ethical Committee of Xijing Hospital (KY20222037-C-1). The trial was registered on clinicaltrials.gov (NCT: NCT05423990). Informed written consent was obtained from all participants or caregivers.

### 2.2. Measurements

#### 2.2.1. Seizure severity evaluation

The Chinese version 2.0 of Liverpool Seizure Severity Scale (LSSS2.0) (14) was applied to evaluate seizure severity of patients (15). No seizures in past 4 weeks was scored as 0 and regarded seizure-free. Ictal scores ranging from 1 to 50 and ≥50 were categorized as mild and severe seizure, respectively.

#### 2.2.2. Anxiety symptoms recording

The HAMA was used for the evaluation of anxiety of patients. The HAMA scores ≥14 indicated the emergence of anxiety symptoms and <14 asymptomatic or possible emergence of anxiety symptoms.

#### 2.2.3. Questionnaire

A questionnaire involving 42-multiple-choice questions and 2 open-ended questions was used, which belonged to the following five parts:

1. Demographic characteristics: sex, age, marital status, educational level, average household monthly income, medical insurance, residence.
2. Epileptic characteristics: seizure severity (12-item LSSS 2.0), course of epilepsy, seizure frequency, ASM therapy, ASM adverse reaction, family history of epilepsy.
3. Attitudes towards SUDEP disclosure: Do you want to get more information about SUDEP? May the SUDEP disclosure to you be a psychological burden? Does the SUDEP disclosure more beneficial than detrimental to you? Should the SUDEP be disclosed to your relatives or caregivers only?
4. Two open-ended questions: What benefits do you think the patients will get if they were informed of SUDEP? What risks do you think the patients will get if they were informed of SUDEP?
5. Assessment of anxiety symptoms (14-item HAMA).

### 2.3. Statistical analysis

Analysis of variance (ANOVA) and dependent samples *t*-test were used to compare the HAMA scores (continuous variable) at baseline, T1 and T2. The counts and percentages of descriptive variables including participants’ demographic characteristics, epileptic characteristics and attitudes towards SUDEP disclosure were calculated. Pearson *χ*^2^ test and Wilcoxon rank sum test were performed to compare the categorical and ordinal variables, respectively. For logistic regression analysis, the univariate test was carried out first to screen out variables related to anxiety with *p* < 0.2 being the prerequisite, which was used for a multivariate logistic regression analysis with the R-package *autoReg*. All statistical analyses were performed using R v.4.0.3.

## 3. Results

### 3.1. Characteristics of study population

From 305 participants, 73 were excluded, because of: prior information and awareness (*n* = 25, 8.2%), cognitive dysfunction (*n* = 7, 2.3%), diagnosed psychiatric comorbidities (*n* = 10, 3.3%), subthreshold symptoms (*n* = 40, 13.1%) and unable to complete survey (*n* = 4, 1.3%). Some PWE fulfill multiple exclusion criteria. Totally, 232 PWE completed the screening evaluation and survey for the questionnaire and we completed a follow-up study of 171 (61 participants dropped out due to phone disconnection or unavailability of internet communication tools). The internal consistency of LSSS 2.0 and HAMA were 0.90 and 0.93, respectively.

Their demographic characteristics, epileptic characteristics and attitudes towards SUDEP disclosure were summarized in Table 1. One hundred (43.1%) were females and 203 (87.5%) were adults aged 18–60 years. There were 105 (45.3%) participants being seizure-free or with mild seizure and 127 (54.7%) with severe seizure. The course of epilepsy was <1 year in 27 (11.6%) participants, 1–10 years in 129 (55.6%) and >10 years in 76 (32.8%). 226 (97.4%) participants have ASM and 217 (93.5%) denied family history of epilepsy. Of all the 232 individuals, 181 (78.0%) would receive information about SUDEP, and 119 (51.3%) complained of psychological burden following the SUDEP disclosure. Meanwhile, 175 (75.4%) believed the SUDEP disclosure was beneficial for themselves, 147 (63.4%) insisted the SUDEP disclosure being limited to their relatives and caregivers only. Furthermore, 127 (54.7%) reached the cutoff of anxiety (HAMA score ≥14), including 113 (48.7%) with anxiety, 8 (3.4%) with obvious anxiety and 6 (2.6%) with severe anxiety (16).

**Table 1:** Participants’ demographic characteristics, epileptic characteristics and attitudes towards SUDEP disclosure associated with anxiety.

Characteristics	Anxiety (%)		*p*-value
	Yes (*n* = 127)	No (*n* = 105)	
Sex			0.945
Male	72 (56.7%)	60 (57.1%)	
Female	55 (43.3%)	45 (42.9%)	
Age			0.441
<18 years	16 (12.6%)	8 (7.6%)	
18–30 years	57 (44.9%)	55 (52.4%)	
30–60 years	52 (40.9%)	39 (37.1%)	
>60 years	2 (1.6%)	3 (2.9%)	
Marital status			0.763
Single	62 (48.8%)	55 (52.4%)	
Married	58 (45.7%)	46 (43.8%)	
Divorced or widowed	7 (5.5%)	4 (3.8%)	
Educational level			0.332
Primary school/secondary school	71 (55.9%)	52 (49.5%)	
Diploma above	56 (44.1%)	53 (50.5%)	
Average household monthly income in RMB			0.351
<3,000	57 (44.9%)	43 (41.0%)	
3,000–6,000	52 (40.9%)	50 (47.6%)	
6,000–10,000	16 (12.6%)	8 (7.6%)	
>10,000	2 (1.6%)	4 (3.8%)	
Medical insurance			0.144
NCMI	61 (48.0%)	37 (35.2%)	
URBMI	35 (27.6%)	37 (35.2%)	
UEBMI	31 (24.4%)	31 (29.5%)	
Residence			0.767
Villages and towns	69 (54.3%)	55 (52.4%)	
City	58 (45.7%)	50 (47.6%)	
Seizure severity			**0.021**
Seizure-free/mild	59 (44.0%)	46 (46.9%)	
Severe	75 (56.0%)	52 (53.1%)	
Course of epilepsy			0.327
0–1 year	16 (12.6%)	11 (10.5%)	
1–10 years	65 (51.2%)	64 (61.0%)	
More than 10 years	46 (36.2%)	30 (28.6%)	
Seizure frequency			**0.037**
<1 per year	48 (37.8%)	53 (50.5%)	
1–11 times per year	43 (33.9%)	32 (30.5%)	
≥12 times per year	36 (28.3%)	20 (19.0%)	
ASM therapy			0.820
No ASM	4 (3.1%)	2 (1.9%)	
Monotherapy	39 (30.7%)	34 (32.4%)	
Polytherapy	84 (66.1%)	69 (65.7%)	
ASM adverse reaction			0.866
No	98 (77.2%)	82 (78.1%)	
Yes	29 (22.8%)	23 (21.9%)	
Family history of epilepsy			0.337
No	117 (92.1%)	100 (95.2%)	
Yes	10 (7.9%)	5 (4.8%)	
Do you want to get more information about SUDEP			0.730
No	29 (22.8%)	22 (21.0%)	
Yes	98 (77.2%)	83 (79.0%)	
May the SUDEP disclosure to you be a psychological burden			0.763
No	63 (49.6%)	50 (47.6%)	
Yes	64 (50.4%)	55 (52.4%)	
Does the SUDEP disclosure more beneficial than detrimental to you			0.582
No	33 (26.0%)	24 (22.9%)	
Yes	94 (74.0%)	81 (77.1%)	
Should the SUDEP be disclosed to your relatives or caregivers only			**<0.001**
No	30 (23.6%)	55 (52.4%)	
Yes	97 (76.4%)	50 (47.6%)	

SUDEP, sudden unexpected death in epilepsy; RMB, Renminbi; NCMI, new rural cooperative medical insurance; URBMI, urban resident basic medical insurance; UEBMI, urban employee basic medical insurance; ASM, anti-seizure medication. Bold font indicates significant results.

Answers to the two open-ended questions were summarized in Table 2. The top benefit associated with SUDEP disclosure for the patients was comprehensive understanding of their own condition, and the top risk was anxiety and depressive mood.

**Table 2:** Description of two open-ended questions about benefits and risks of SUDEP disclosure.

What benefits do you think patients will get if they were informed of SUDEP	*N* (%)
Getting a comprehensive understanding of your own condition	192 (82.8%)
Getting a better prognosis	127 (54.7%)
Increasing your understanding and trust in treating neurologist	120 (51.7%)
Getting a better medication compliance	72 (31.0%)
Others	9 (3.9%%)
None	12 (5.2%)

What risks do you think patients will get if they were informed of SUDEP	*N* (%)
Causing anxious or depressed mood	149 (64.2%)
Causing psychological burden to induce seizures	120 (51.7%)
Getting a poor prognosis	40 (17.2%)
Exaggerating potential risks undermines mutual understanding and trust	26 (11.2%)
Others	14 (6.0%)
None	42 (18.1%)

SUDEP, sudden unexpected death in epilepsy.

**Figure 3:**
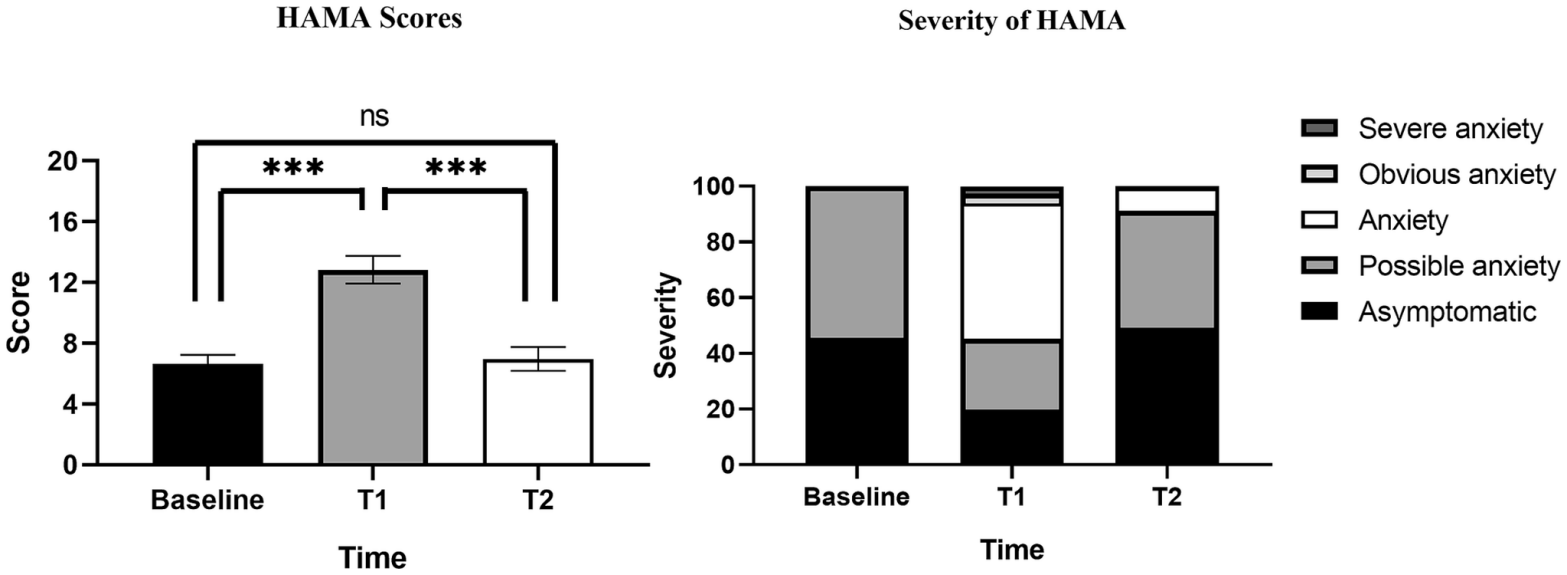
HAMA scores and severity at baseline, T1 and T2.

HAMA scores at baseline, T1, T2 were summarized in Table 3. The results of Table 3 are visualized in Figure 3. The HAMA scores of 232 PWE were 6.64 ± 4.54, 12.84 ± 7.00 and 6.96 ± 5.25 at baseline, T1 and T2. HAMA scores at T1 were significantly higher than at baseline and T2, while there was no statistical difference between baseline and T2. Compared with 127 PWE with HAMA scores ≥14 at T1, Only 15 PWE had HAMA scores ≥14 after 4 weeks of follow-up at T2. Of the 14 PWE whose severity of HAMA were classified as obvious or severe anxiety, only 1 PWE had obvious anxiety at T2.

**Table 3:** Participants’ score and severity of HAMA at baseline, immediately at the end of SUDEP disclosure (T1) and 4 weeks after the end of SUDEP disclosure (T2).

(A)			
	Mean ± SD, CI		
HAMA	Baseline (*n* = 232)	T1 (*n* = 232)	T2 (*n* = 171)
Score	6.64 ± 4.54 (6.05–7.22)	12.84 ± 7.00 (11.93–13.74)	6.96 ± 5.25 (6.17–7.76)

Severity	*n* (%)		
Asymptomatic	106 (45.7%)	46 (19.8%)	84 (49.1%)
Possible anxiety	126 (54.3%)	59 (25.4%)	72 (42.1%)
Anxiety	0 (0.0%)	113 (48.7%)	14 (8.2%)
Obvious anxiety	0 (0.0%)	8 (3.4%)	1 (0.6%)
Severe anxiety	0 (0.0%)	6 (2.6%)	0 (0.0%)

(B)		
	*N* (%)	*p*-value
HAMA score	Baseline (*n* = 232)	**<0.001**
	T1 (*n* = 232)	
	T2 (*n* = 171)	
	Baseline (*n* = 232)	**<0.001**
	T1 (*n* = 232)	
	T1 (*n* = 232)	**<0.001**
	T2 (*n* = 171)	
	Baseline (*n* = 232)	0.612
	T2 (*n* = 171)	

SUDEP, sudden unexpected death in epilepsy; HAMA, Hamilton anxiety rating scale; T1, time point 1; T2, time point 2; SD, standard deviation; CI, confidence interval. Bold font indicates significant results.

### 3.2. Univariate analysis and multivariate logistic regression

**Figure 4:**
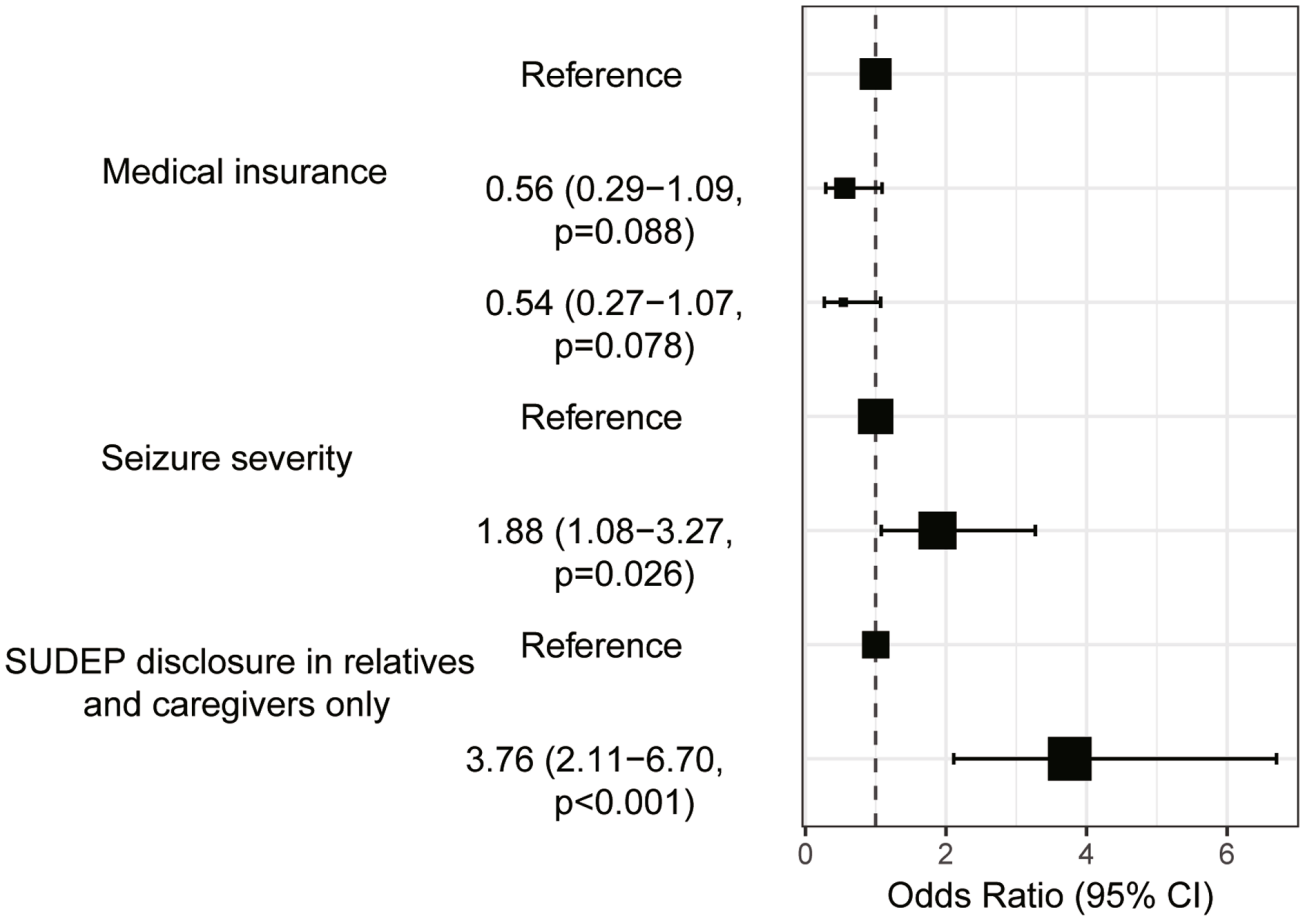
Forest map of Multivariate logistic regression.

Univariate analysis found four variables with *p* < 0.2 being included in multivariate logistic regression, namely participants’ medical insurance, seizure severity, seizure frequency, and should the SUDEP be disclosed to your relatives or caregivers only. Multivariate logistic regression confirmed the medical insurance, seizure severity and should the SUDEP be disclosed to your relatives or caregivers only as independent risk factors of anxiety in PWE after SUDEP disclosure (Table 4). The results of Table 4 are visualized in Figure 4. For example, PWE with urban resident basic medical insurance (URBMI) and urban employee basic medical insurance (UEBMI) were 0.56 and 0.54 times more likely to experience anxiety after SUDEP disclosure than those with new rural cooperative medical insurance (NCMI). PWE with severe seizure severity were 1.88 times more likely to experience anxiety than those with favorable seizure control. Specifically, for PWE with severe seizure severity, 75 (59.0%) experienced anxiety and 52 (41.0%) reported no anxiety symptoms. In contrast, for PWE with seizure-free or mild seizure severity, 59 (56.2%) experienced anxiety and 46 (43.8%) reported no such response. PWE insisting the SUDEP disclosure being limited to their relatives and caregivers were 3.76 times more likely to suffer anxiety than those not, with 97 (66.0%) of the former and 30 (35.3%) of the latter group evincing anxiety, respectively.

**Table 4:** Univariable and multivariate logistic regression analysis to see the predictors of anxiety in epilepsy patients after SUDEP disclosure.

		OR (univariable)	OR (multivariable)	OR (final)
Medical insurance	NCMI			
	URBMI	0.57 (0.31–1.06, *p* = 0.077)	0.56 (0.29–1.09, *p* = 0.088)	0.56 (0.29–1.09, *p* = 0.088)
	UEBMI	0.61 (0.32–1.15, *p* = 0.128)	0.54 (0.27–1.07, *p* = 0.078)	0.54 (0.27–1.07, *p* = 0.078)
Seizure severity	Seizure-free/mild			
	Severe	1.85 (1.10–3.12, *p* = 0.021)	1.88 (1.08–3.27, *p* = 0.026)	1.88 (1.08–3.27, *p* = 0.026)
Seizure frequency	<1 per year			
	1–11 times per year	1.48 (0.81–2.71, *p* = 0.199)	1.45 (0.76–2.76, *p* = 0.258)	
	≥12 times per year	1.99 (1.02–3.89, *p* = 0.045)	1.38 (0.66–2.88, *p* = 0.392)	
Whether you support SUDEP disclosure in your relatives and caregivers only	No			
	Yes	3.56 (2.03–6.23, *p* < 0.001)	3.66 (2.04–6.56, *p* < 0.001)	3.76 (2.11–6.70, *p* < 0.001)

SUDEP, sudden unexpected death in epilepsy; OR, odds ratio; NCMI, new rural cooperative medical insurance; URBMI, urban resident basic medical insurance; UEBMI, urban employee basic medical insurance.

## 4. Discussion

This was the first multi-center study on SUDEP disclosure to PWE in China to our knowledge. Of the 305 PWE, only 8.2% reported SUDEP disclosure and awareness, which is lower than the proportion of 14.3% to 43% in other countries (12, 13). A possible explanation for this difference might be the lack of SUDEP awareness and education among neurologists and PWE (17, 18). However, the concerns about the possible undue anxiety in patients after SUDEP disclosure may also partly account for the situation (7, 8, 10). So far, it has not been concluded whether the SUDEP disclosure induces anxiety in PWE. Although the anxiety symptoms occurred in more than half of participants in the present investigation, several other studies failed to reveal negative sentiment induced by SUDEP disclosure (11, 12). It has been a long-running debate whether all PWE and their caregivers should be informed of SUDEP. Recent studies advocated all PWE were counseled with SUDEP as part of education to increase awareness of the risks and precautions (19–21). The 2017 SUDEP practice guideline also recommended SUDEP disclosure for optimizing epilepsy management (6). However, for low-risk PWE with proper seizure control, particularly without GTCS, the negative effects of SUDEP disclosure might outweigh possible benefits (7, 8, 10, 22), consistent with the top patient-identified risk of SUDEP disclosure in this study. The seizure severity and whether to disclose SUDEP to PWE were the two independent risk factors for anxiety after SUDEP disclosure. So the relevant countermeasures might mitigate anxiety caused by SUDEP disclosure.

Our study found that although SUDEP disclosure caused initial short-term acute anxiety in PWE, the acute anxiety gradually disappeared and returned to their original state during subsequent follow-up. Among PWE with a score ≥14 at T1, very few (11.0%) were obvious anxiety or severe anxiety, with only mild increases in HAMA scores in the vast majority of PWE. In the long term, SUDEP disclosure had no effect on the mood of PWE, and no risk of chronic anxiety was found, which is consistent with what had been reported in previous studies (11, 12, 23–25). Even PWE who were assessed as severe or obvious anxiety at T1 showed significant reductions in HAMA scores at T2. We feel that the findings may help to address the neurologists’ concerns that SUDEP disclosure possibly causes anxiety in PWE and also to increase the proportion of SUDEP disclosure.

In the study, it was found that PWE with NCMI had a higher risk of acute anxiety after SUDEP disclosure compared to those with URBMI and UEBMI. The underlying reasons why the type of medical insurance influenced the level of anxiety after SUDEP disclosure among PWE remained for further research. One possible explanation is that people from rural areas may have less access to information about complications of epilepsy than urban people. This suggests that current availability and consistency of public health education for PWE may vary and remain uneven across different regions.

PWE with severe seizure were more likely to experience acute anxiety in our study. Some other reports identified SUDEP may occur in early or benign epilepsy (26, 27). Therefore, it may be inadequate to just rely on disease course or seizure severity to determine whether to bring about SUDEP disclosure. Currently, the reasonable time for the PWE to be informed of SUDEP should be at first diagnosis or early course of the disease (20). Given the risk of anxiety, neurologists should be more considerate when delivering the information to severe PWE. However, that does not mean not talking about SUDEP.

According to our result, the SUDEP should be disclosed to PWE rather than just to their caregivers. However, an Italian survey indicated that this behavior conflicted with clinical practice guidelines (9). Neurologists also choose not to inform the patients for fear of the excessive anxiety which may exacerbate the condition of epilepsy (9). In the present study, 147 (63.4%) participants would prefer the SUDEP being disclosed to their relatives and caregivers only. The reasons behind this choice remains to be characterized and may relate to the concerns about poor prognosis and bad clinical outcome. Direct SUDEP disclosure in PWE not only asserts their right to know the information but also avoids the risk of undue anxiety.

Establishing a patient-centered communication strategy may help enhance the proportion of SUDEP disclosure to patients as well as address neurologists’ concerns about negative effects. A Canada study has shown that the PWE would like to be informed of SUDEP, especially when the SUDEP disclosure was scheduled on a case-by-case basis (12). Our study verified the majority of PWE (78.0%) still embrace more information about SUDEP following preliminary disclosure. So despite the possible negative effects, we would recommend the SUDEP disclosure to patients along with the first diagnosis. We favor that the communication about SUDEP begins soon after diagnosis to build a bilateral trust between patients and physician. Our study indicates that the SUDEP disclosure would not cause undue anxiety when symptoms of the PWE got improved or the seizures controlled. For those with severe seizures or high risk of SUDEP, neurologists should focus on the seizure control while weighing the risks and benefits of any new approach (6). SUDEP disclosure may be a priority especially in these cases. Direct disclosure and effective epilepsy therapies act to avoid or minimize negative effect of SUDEP disclosure, which depends on a good bilateral relationship and full trust between neurologists and patients. However, till now, there is still no consensus on a standardized approach about SUDEP disclosure. A gap between the right of PWE and caregivers to know the truth and the neurologists’ willingness for SUDEP disclosure implies a lack of education and training on such issue in medical care (28). Finally, it is also an important measure to call on the government and health institutions to increase the investment in medical insurance for PWE.

This study has several limitations. First, we excluded PWE with a history of diagnosed psychiatric comorbidities or subthreshold symptoms and surveyed the vast majority of participants with course of epilepsy longer than 1 year. However, as we did not include PWE with anxiety symptoms, therefore, the response of these patients to SUDEP disclosure and their willingness to receive disclosure still need to be further studied. Second, we only focused on the effect of SUDEP disclosure on participants’ mood. The long-term effects of SUDEP disclosure on lifestyle, medication adherence and preventive measures in PWE still needs to be clarified. Third, participants in our study were all from Chinese tertiary epilepsy center, and most of participants were aged 18–60 years. Due to cultural and age differences, results of this study may not be generalizable to other populations.

## 5. Conclusion

In conclusion, we surveyed the association of anxiety with SUDEP disclosure in PWE in this survey study. SUDEP disclosures may cause short-term acute anxiety, but have no long-term effects in PWE. Results of the present study reveal that acute anxiety caused by SUDEP disclosure may be more common in PWE with NCMI and severe seizures. Meanwhile, direct SUDEP disclosure to PWE may lower risk of acute anxiety compared with just to their relatives and caregivers. If a neurologist decides to disclose SUDEP, establishing a patient-centered communication strategy to avoid negative effect are recommended.

## Data availability statement

The raw data supporting the conclusions of this article will be made available by the authors, without undue reservation.

## Ethics statement

The studies involving humans were approved by the Medical Ethical Committee of Xijing Hospital (KY20222037-C-1). The studies were conducted in accordance with the local legislation and institutional requirements. The participants provided their written informed consent to participate in this study.

## Author contributions

YP: Formal analysis, Investigation, Methodology, Writing – original draft. GH: Writing – original draft. ZeW: Writing – original draft. NY: Writing – original draft. ZiW: Writing – original draft. XL: Writing – original draft. XH: Writing – original draft. JW: Writing – original draft. XZ: Writing – original draft. ZC: Writing – original draft. SQ: Writing – original draft. JB: Conceptualization, Methodology, Writing – review & editing. YL: Conceptualization, Methodology, Resources, Writing – review & editing.
